# Lessons Learned From the Introduction of Inactivated Poliovirus Vaccine in Bangladesh

**DOI:** 10.1093/infdis/jiw510

**Published:** 2017-02-04

**Authors:** Concepcion F Estivariz, Cynthia J Snider, Abhijeet Anand, Lee M Hampton, Tajul I Bari, Mallick M Billah, Shua J Chai, Steven G Wassilak, James D Heffelfinger, K Zaman

**Affiliations:** 1 Global Immunization Division, Atlanta, Georgia; 2 Division of Global Health Protection, Center for Global Health, Centers for Disease Control and Prevention, Atlanta, Georgia; 3 Expanded Programme on Immunization, Dhaka, Bangladesh; 4 Field Epidemiology Training Program Bangladesh, Institute of Epidemiology, Disease Control and Research, Ministry of Health and Family Welfare, Dhaka, Bangladesh; 5 International Centre for Diarrhoeal Diseases Research, Dhaka, Bangladesh

**Keywords:** polio, routine immunization, inactivated poliovirus vaccine, new vaccine introduction

## Abstract

**Background:**

We assessed programmatic adaptations and infants' uptake of inactivated poliovirus vaccine (IPV) after its introduction into the routine immunization schedule in Bangladesh.

**Methods:**

Using convenience and probability sampling, we selected 23 health facilities, 36 vaccinators, and 336 caregivers, within 5 districts and 3 city corporations. We collected data during August–October 2015 by conducting interviews, reviewing vaccination records, and observing activities.

**Results:**

Knowledge about IPV was high among vaccinators (94%). No problems with IPV storage, transport, or waste disposal were detected, but shortages were reported in 20 health facilities (87%). Wastage per 5-dose vaccine vial was above the recommended 30% in 20 health facilities (87%); all were related to providing <5 doses per open vial. Among eligible infants, 87% and 86% received the third dose of pentavalent and oral poliovirus vaccine, respectively, but only 65% received IPV at the same visit. Among 73 infants not vaccinated with IPV, 58% of caregivers reported that vaccine was unavailable.

**Conclusions:**

Bangladesh successfully introduced IPV, but shortages related to insufficient global supply and high vaccine wastage in small outreach immunization sessions might reduce its impact on population immunity. Minimizing wastage and use of a 2-dose fractional-IPV schedule could extend IPV immunization to more children.

In accordance with the polio endgame plan developed by the Global Polio Eradication Initiative [1, 2], Bangladesh introduced inactivated poliovirus vaccine (IPV) into its routine immunization (RI) schedule on 21 March 2015 [3]. The National Committee on Immunization Practices of Bangladesh supported the introduction of IPV as a single, stand-alone dose to be administered at 14 weeks of age together with the third dose of oral poliovirus vaccine (OPV) and pentavalent vaccine (protecting against diphtheria, tetanus, pertussis, hepatitis B, and *Haemophilus influenza* type b infection), as recommended by the World Health Organization (WHO) [4, 5]. The pneumococcal conjugate vaccine (PCV) was introduced simultaneously with IPV, as 3 doses administered at 6, 10, and 18 weeks of age [3]. The 18-week visit for the third dose of PCV (PCV3) was added to the schedule, to avoid administration of >2 injectable vaccines during one visit.

The introduction of new vaccines can have a positive effect on health systems in areas where technical guidance and adequate financing are available, but insufficient planning can further compromise already weak immunization and health systems [6, 7]. Because Bangladesh was among the first low-resource, OPV-using countries to introduce IPV in RI services, we conducted an early assessment to guide the country's immunization program and to identify lessons useful to countries introducing IPV. The primary objectives were to assess (1) programmatic adaptation to IPV introduction; and (2) knowledge, attitudes, and practices of vaccinators and caregivers related to IPV. A secondary objective was to assess the effect of IPV introduction on delivery of other vaccines through RI services. Issues related to cold chain are presented in a separate report in this supplement.

## METHODS

### Background: Coordination of Routine Immunization Services in Bangladesh

Bangladesh is administratively divided into 7 divisions with 64 districts and 11 large urban centers, known as city corporations (CCs). The estimated population for 2015 was 160 million, with a birth cohort of approximately 3.5 million [8]. A national coordinator from the Ministry of Health and Family Welfare oversees the Expanded Program for Immunization (EPI) countrywide, but management at lower administrative levels is different for districts and CCs. Districts are divided into *upazilas* (subdistricts), unions, wards, and blocks. For management of the EPI, each ward is further divided into 8 subblocks, each with a population of about 1000. Each subblock is visited once monthly by local health workers for provision of RI services in outreach sites. EPI staff in health facilities at the ward or *upazila* level coordinate outreach RI delivery and may provide RI services on-site 2–3 times weekly [9].

CCs are subdivided into zones and wards. Vaccine supply is managed by the national EPI, but delivery of services is coordinated by local CC government staff and nongovernmental organizations. Single or multiple nongovernmental organizations may provide RI services in the same ward through outreach vaccination sites or exclusively at health clinics.

Standardized forms and logbooks are used to track vaccines and supplies at the national, district, and health facility levels. The monthly vaccination report is used to record the target population for each month, the number of infants who have received each dose of each antigen, the total number of vials used for each antigen, and the proportion of doses wasted. The immunization registry book, maintained by a health facility or outreach vaccination site, tracks vaccine and doses administered to each child born in the catchment area. The immunization card that each child receives upon their first contact with the system contains the dates when each vaccine dose is received.

### Sample Selection

The assessment sample was selected using a modification of the methods suggested by the WHO for evaluating vaccines after their introduction [10]. We purposefully selected 5 districts and 3 CCs to achieve representation across a variety of criteria of program performance and access, in terms of (1) immunization services primary management (EPI or CC); (2) estimated 2013 coverage for the third dose of pentavalent vaccine (Penta3; ie, low [<85%], medium [85%–95%], or high [≥95%]); (3) the presence of difficult-to-reach populations, such as geographically isolated groups or ethnic minorities; and (4) geographical distribution throughout the country.

In districts (under EPI management), wards were stratified as urban, rural, or having difficult-to-reach populations, and 1–2 wards per strata were randomly selected to obtain a maximum of 3 wards per district. In CCs, 3 wards were randomly selected without stratification. Within each ward, trained surveyors visited the health facility (at the *upazila* or ward level) responsible for storing vaccines and coordinating immunization services, as well as 2 outreach vaccination sites. Overall, 24 health facilities and 48 outreach sites were targeted. EPI managers at the national and district/CC levels were also interviewed about preparation activities.

The surveyors also conducted interviews among caregivers residing in the catchment areas of outreach vaccination sites. Caregivers of children born 1 January–30 April 2015 (ie, those old enough to have received IPV at the time of interview) were eligible to participate in the survey. Using 2014 Bangladesh population and annual birth rate figures [8], it was estimated that 7 children per approximately 1000 residents would be of the targeted age range. To account for population variability, we set a target of 300–330 interviews with a maximum of 7 interviews per community. Immunization registry books and information provided by local health workers and community volunteers were used to identify residences of eligible caregivers.

### Activities and Tools Used for the Assessment

The assessment included interviews (immunization managers, vaccinators, and caregivers), records review, and on-site observation of immunization activities. Paper-based standardized questionnaires and observation checklists were used for data collection (Table 1). Before conducting interviews, surveyors obtained verbal informed consent from immunization managers and vaccinators, and written informed consent from caregivers. The questionnaires and checklists used to interview staff and review records were in English; caregiver questionnaires and informed consent forms were in Bangla.

**Table 1.** Activities Conducted and Tools Used for the Assessment at Each Health Administrative Level

Health Level	Activities Performed	Tools Used and Information Collected
Health facility responsible for vaccine storage and distribution at the ward	Interviewed staff in charge of immunization; completed observation checklist; collected data from monthly vaccination reports for January–July 2015	Questionnaire and check-list: immunizations and vaccine management (immunization services provided, vaccine stock management, dry storage, and waste management); review of monthly vaccination reports (target population and number of vaccine doses received, used, and wasted)
Outreach vaccination session in a ward	Interviewed vaccinators; completed observation checklist of vaccination activities and supplies; interviewed caregivers of infants aged 5–8 mo residing in the catchment area	Questionnaire: vaccinator (training, knowledge, attitudes, and practices related to administration of IPV and of multiple injectable vaccines in 1 visit); questionnaire: caregiver in the community (vaccines received by the infant, reasons for missing any vaccine, and caregiver's knowledge and attitudes about IPV and infant's receiving several injectable vaccines in 1 visit)

Administrative data on the number of vaccine doses received, administered, and wasted were obtained from the monthly vaccination reports maintained in the health facilities. Data were entered directly into an Excel spreadsheet to allow rapid calculation of coverage and wastage. Because IPV introduction was staggered in the sampled areas between 21 March 2015 and 11 April 2015 and because the evaluation was conducted during August–September 2015, data abstracted from vaccination records were limited to those collected 3 months before IPV introduction (January, February, and March) and 3 months after introduction (May, June, and July).

The fieldwork was conducted by 5 teams consisting of 1 or 2 medical officers and a field research officer. After pilot testing and revision of data collection instruments, fieldwork was completed between 17 August and 11 October 2015. Teams entered data from paper forms into a Microsoft Access database daily. Investigators used Excel and SAS 9.3 to perform descriptive analyses, calculating frequencies, percentages, medians, means, and ranges. Measures of precision were not calculated and statistical testing not conducted because nonprobability sampling was used. The study was approved by the research and ethical review boards of the International Centre for Diarrhoeal Diseases Research. The Centers for Disease Control and Prevention determined the project to be a public health program evaluation and not human subjects research.

## RESULTS

### Interview With the National Coordinator of the EPI on the Preparations for IPV Introduction

IPV introduction was financed with a Gavi grant that funded vaccine purchase through the United Nations Children's Emergency Fund at $0.80 per child vaccinated. The country cofinanced $0.20 per dose, and additional funding from international partners (UNICEF and WHO) was used to complete staff training and printing of communication materials. Country officials had requested supplies of IPV in 1- or 2-dose vials because they anticipated high wastage with 5-dose and 10-dose vials because of the small size of many outreach vaccination sessions [11]. Because of limited global supplies of both the 1- and 2-dose presentations, Bangladesh received 5-dose vials, with the total amount provided being enough for administration of 1 dose per child of eligible age, assuming 30% wastage for each vial.

Other activities conducted in preparation for IPV introduction included (1) development of an addendum to the country's immunization guidelines, with information about the new vaccines; (2) updating and printing forms and registries used to manage vaccine distribution and collect vaccination data; (3) preparation of training materials and provision of cascade training during January and February 2015; and (4) promotion of the new vaccines through the mass media, communications to medical professional bodies, posters, and brochures.

### Observations, Records Review, and Interviews With Immunization Managers at Health Facilities

The teams visited 23 health facilities that provided RI services for the 24 wards selected for the assessment (in 1 district, 2 wards were covered by the same health facility). Fourteen health facilities were managed by EPI and 9 by CC immunization programs. Twenty-two health facilities (96%) administered vaccines through outreach vaccination sessions, for an average of 16.5 infants per session (range, 2–59 infants per session; Table 2).

**Table 2.** Information From Interviews With Immunization Managers and Observation of Activities and Equipment at 23 Health Facilities

Variable	Value
Type of vaccination activities	
Facilities with vaccination services on-site	20 (87)
Days per week with on-site vaccination sessions	2 (2–6)
Children vaccinated on-site per session	10.0 (6.5–16.5)
Facilities with outreach vaccination sessions	22 (96)
Children vaccinated per outreach session	12.5 (10–18)
Availability of updated vaccination documents that included IPV and PCV	
National immunization guidelines	13 (57)
Monthly vaccine report	23 (100)
Vaccine distribution registry	14 (61)
Newborn registration form	7 (30)
Reported shortages of vaccines or supplies	
IPV	16 (69)
Other vaccines (measles vaccine and/or BCG)	9 (39)
Immunization supplies (sharp disposal boxes, syringes, immunization cards)	9 (39)
Observation of rooms for storage of vaccines and immunization supplies	
Adequate storage space available	20 (87)
Supplies kept under clean and dry conditions	21 (91)
Space well organized, with supplies easily accessible	22 (96)
Found stored IPV vials with VVM in stage III–IV^a^	0 (0)
Waste management practices and observation of facilities	
Mechanisms used for sharps disposal by facility	
Burial	8 (35)
Burning and burial	5 (22)
Shipment to another location for disposal	10 (43)
Immunization managers reported problems with waste disposal after IPV introduction	0 (0)
Observation of discarded vials/supplies in the premises	3 (13)
Inadequate fencing or closing of waste disposal site	15 (65)

Data are no. (%) of respondents or median value (interquartile range).

Abbreviations: BCG, bacillus Calmette-Guerin; IPV, inactivated poliovirus vaccine; PCV, pneumococcal conjugate vaccine; VVM, vaccine vial monitor.

^a^ Stage III–IV in the VVM on a vaccine vial indicates exposure to heat long enough to have affected vaccine potency and requires prompt disposal of that vial. VVM was not observed in 3 facilities that had stocked out of IPV.

All immunization managers reported that staff received IPV training before the introduction. The national immunization guidelines were available in 19 of 23 health facilities (83%), but only 13 (68%) had guidelines updated with IPV information. The updated monthly vaccination report was available in all health facilities, but other forms were updated less frequently (Table 2).

All 23 health facilities received 5-dose IPV vials with vaccine vial monitors on top of the vials, which were required by the manufacturer's instructions and Bangladesh's EPI guidelines to be discarded within 6 hours of opening. All managers reported that these guidelines were followed.

Shortages of IPV between April and the time of the visit were reported in 69% of health facilities, with a mean shortage duration of 5.3 weeks (range, 2–11 weeks). Shortages occurred during May–July in facilities from 4 districts and during August–September in facilities from 3 districts. All facilities cited insufficient supplies provided for their requirements. Shortages of bacillus Calmette-Guerin– and/or measles virus–containing vaccine were reported by 39% of health facilities; none reported scarcity of PCV or pentavalent vaccine (Table 2). Data abstracted from monthly vaccination reports showed that, on average, health facilities received 66%, 46%, and 39% of doses requested in May, June, and July, respectively. During the same period, monthly wastage per vial ranged between 5% and 80%, and the 3-month average was >30% in 20 health facilities (87%). Wastage was related to opened vials in which <5 children were vaccinated; 0 wastage was reported for inadequate handling of unopened vials (eg, breakage and cold chain mismanagement).

**Figure 1.**
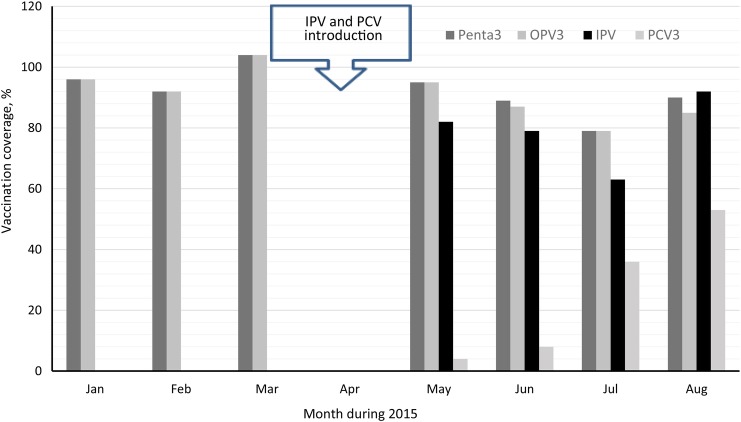
Average vaccination coverage per month for the third doses of pentavalent vaccine (Penta3), oral poliovirus vaccine (OPV3), and pneumococcal conjugate vaccine (PCV3) and for inactivated poliovirus vaccine (IPV) in 23 health facilities, Bangladesh, 2015. Coverage was calculated by dividing the number of children who had received each vaccine dose in a particular month by the number of children who were expected to receive any vaccine per month. IPV and PCV were introduced between 21 March and 11 April in the health facilities assessed. Data from April were not collected.

Monthly administrative coverage for IPV and the third dose of pentavalent vaccine, OPV, and PCV during January–July (excluding April) is shown in Figure 1. The 3-month average coverage for the third doses of pentavalent vaccine and OPV was 97% during January–March, whereas during May–July it only reached 88% and 87%, respectively. Immunization managers reported that monsoon rains and Ramadan-related reduction in daily activities and travel usually decreased immunization activities during May–July. Coverage for IPV during May–July was 74%. Coverage for the third dose of PCV, based on administrative data, was low because the denominators had not been restricted to include only children who had started the PCV series (Figure 1).

Storage space for supplies was adequate, and waste-disposal logistics were not affected by the additional load, although several health facilities did not follow waste-disposal guidelines appropriately (Table 2).

### Interviews With Vaccinators

Interviews with 36 vaccinators were conducted in the 48 outreach vaccination sites because the same vaccinator was responsible for several or all outreach immunization sites in some wards. Thirty-three of vaccinators (92%) received training on IPV before its introduction, and 94% correctly answered questions about IPV handling and delivery (Table 3). Only 1 vaccinator (4%) was observed administering an injectable vaccine by use of a wrong technique (IPV injection in the lateral instead of the anterior aspect of the thigh).

**Table 3.** Vaccinators' Knowledge and Practices Regarding Administration of Inactivated Poliovirus Vaccine (IPV) and Multiple Injectable Vaccines in 1 Visit

Variable	Vaccinators, Proportion (%)		
	EPI Districts	City Corporations	Total
Vaccinator's training and knowledge about IPV			
Received training before IPV introduction	17 (94)	16 (89)	33 (92)
Answered correctly age for IPV vaccination	18 (100)	17 (94)	35 (97)
Answered correctly temperature for IPV storage	17 (94)	17 (94)	34 (94)
No errors during administration of injectable vaccine	18 (100)	17 (94)	35 (97)
Vaccinator reported shortage of IPV vials for sessions	15 (83)	11 (61)	26 (72)
No. of children for whom the vaccinator opens a vial of IPV			
1	12 (67)	0 (0)	12 (33)
2	4 (22)	5 (28)	9 (25)
3	2 (11)	8 (44)	10 (28)
4	0 (0)	5 (28)	5 (14)
Vaccinator's action when only 1 child required IPV			
Opened a new vial	12 (67)	0 (0)	12 (33)
Asked to wait at the site until more children arrive	5 (28)	13 (72)	18 (50)
Asked to come back to the next vaccination session	1 (6)	9 (50)	10 (28)
Asked to go to another vaccination site	4 (22)	4 (22)	8 (22)
Vaccinator's degree of comfort with giving ≥3 injections in 1 visit			
Very comfortable	3 (17)	11 (61)	14 (39)
Comfortable	7 (39)	7 (39)	14 (39)
Not comfortable	8 (44)	0 (0)	8 (22)
Vaccinator's actions if an infant aged 5 mo needed PCV, IPV, and pentavalent vaccine			
Give all 3 injectable vaccines at the same visit	0 (0)	3 (17)	3 (8)
Delay giving PCV	15 (83)	9 (50)	24 (67)
Delay giving IPV	3 (17)	6 (33)	9 (25)
Vaccinator's beliefs about caregivers' preferences involving multiple injectable vaccines			
Child could receive 3 injectable vaccines in 1 visit	5 (28)	3 (17)	8 (22)
Child could receive 2 injectable vaccines at 2 visits and 1 injectable vaccine at another visit	7 (39)	13 (72)	20 (56)
Child should receive 1 vaccine per visit over 3 visits	6 (33)	2 (11)	8 (22)

Data are from 36 interviews, with 18 each conducted in EPI districts and city corporations.

Abbreviations: BCG, bacillus Calmette-Guerin; EPI, Expanded Program on Immunization; PCV, pneumococcal conjugate vaccine.

Twenty-six vaccinators (72%) reported shortages of IPV for vaccination sessions because of insufficient supply from the health facility (Table 3). When asked about their usual practices when a single child presented for IPV receipt, one third of vaccinators (67% in EPI districts and 0% in CCs) reported that they opened a new vial, but 50% asked parents of the initial child to wait for more infants to present for IPV receipt, and the rest asked parents to come to another session or go to another site to be vaccinated.

Regarding administration of multiple (ie, >2) injectable vaccines in 1 vaccination visit, 56% of vaccinators in EPI sites and all vaccinators in CCs felt comfortable or very comfortable giving 3 injections during a single visit (Table 3). However, only 3 vaccinators (8%) stated they would give 3 injections in the same visit to a 5-month-old infant who needed PCV, pentavalent vaccine, and IPV. More than half (56%) of all vaccinators (39% in EPI sites and 72% in CC sites) believed that caregivers would prefer that 3 injectable vaccines be administered in 2 visits.

### Interviews With Caregivers in the Community

Of the 385 households with eligible infants, 48 caregivers were not interviewed because they were not at home (40) or refused to participate (4; reasons were not recorded for the remaining 4). After excluding 1 interview that exceeded the targeted 7 interviews per community, 336 caregiver interviews (87%) were included in the analysis. Mothers accounted for 97% of interviewees, and immunization cards were available for 98% of infants.

Overall, 100% and 87% of infants of eligible age received Penta1 and Penta3, respectively; 65% received IPV; 86% received OPV3; and 98% and 55% received PCV1 and PCV3, respectively (Table 4). All children vaccinated with IPV also received Penta3, but 25% of infants (73 of 293) who received Penta3 did not receive IPV during the same vaccination visit.

**Table 4.** Vaccination Status of Infants Participating in the Community Survey, and Proportion Who Had Received Vaccines Appropriate for Their Age per the Routine Immunization Schedule in Bangladesh

Variable	EPI Districts	City Corporations	Total
Infants' age, wk, median (range)	28.0 (16.9–39.6)	28.1 (16.7–39.9)	28.0 (16.7–39.9)
Caregivers with immunization card	205/210 (98)	125/126 (99)	330/336 (98)
Received BCG	209/210 (99)	124/126 (98)	333/336 (99)
Received 3 doses of pentavalent vaccine	182/210 (87)	111/126 (88)	293/336 (87)
Received 3 doses of OPV	177/210 (84)	111/126 (88)	288/336 (86)
Received IPV	121/210 (58)	99/126 (79)	220/336 (65)
Received 3rd dose of pentavalent but not IPV	61/182 (34)	12/111 (11)	73/293 (25)
Received 1st dose of PCV^a^	146/148 (99)	81/83 (98)	227/231 (98)
Received 2nd dose of PCV^a^	137/148 (93)	77/83 (93)	214/231 (94)
Received 3rd third dose of PCV^b^	76/144 (53)	47/80 (59)	123/224 (55)

Data are proportion (%) of infants, unless otherwise indicated.

Abbreviations: BCG, bacillus Calmette-Guerin; EPI, Expanded Program on Immunization; IPV, inactivated poliovirus vaccine; OPV, oral poliovirus vaccine; PCV, pneumococcal conjugate vaccine.

^a^ Denominator restricted to infants who were eligible to start their primary pentavalent vaccine series after PCV introduction on 6 April 2015.

^b^ Denominator restricted to infants who were eligible to start their primary pentavalent vaccine series after PCV introduction on 6 April 2015 and were ≥18 weeks of age at the time of the survey.

Among the 43 infants who missed Penta3, the most common reasons were that the infant was sick (30%) or missed the appointment (19%; Table 5). For the 73 infants who had not received IPV with Penta3, 42 caregivers (58%) said no vaccine was available, and 23 (32%) did not know why their child had not been vaccinated. Of these 23 caregivers, 16 (70%) did not know about the introduction of IPV. No caregivers reported mistrust of IPV or concerns about their child receiving 2 polio vaccines (IPV and OPV3) at the same visit.

**Table 5.** Reasons for Missing the Third Dose of Pentavalent and/or Inactivated Polio Vaccine (IPV) Among Infants in the Community Survey

Reason	Infants, Proportion (%)	
	Missed 3rd Dose of Pentavalent Vaccine	Received 3rd Dose of Pentavalent Vaccine but Not IPV
Caregiver thought child was too sick	13/43 (30)	…
Caregiver missed the appointment	8/43 (19)	…
Time of vaccination unknown	4/43 (9)	…
Delayed start of vaccination series	3/43 (7)	…
Caregiver did not know why	2/43 (5)	23/73 (32)
Vaccine out of stock/shortage	1/43 (2)	42/73 (58)
Caregiver unaware of the need for vaccine	1/43 (2)	6/73 (8)
Other	11/43 (26)	2/73 (2)

About half (48%) of caregivers knew about IPV (Table 6). Among those who were aware of IPV, the major sources of information were health workers (83%), mass media (19%), and posters and pamphlets (7%).

**Table 6.** Caregivers' Knowledge and Attitudes Regarding Inactivated Polio Vaccine (IPV) and Multiple Injectable Vaccines in the Same Visit

Variable	Responding Caregivers, Proportion (%)		
	EPI Districts	City Corporations	Total
Infant's mother	202/210 (96)	123/126 (98)	325/336 (97)
Knew about IPV	101/210 (48)	59/126 (47)	160/336 (48)
Source of information about IPV^a^			
Community worker or vaccinator	76/101 (75)	57/59 (97)	133/160 (83)
Physician	2/101 (2)	0/59 (0)	2/160 (1)
Friend/family	0/101 (0)	0/59 (0)	0/160 (0)
Mass media (television, radio)	30/101 (30)	1/59 (2)	31/160 (19)
Poster or pamphlet	8/101 (8)	3/59 (5)	11/160 (7)
Maximum no. of injections with which caregiver was comfortable^a^			
1	57/210 (27)	13/126 (10)	70/336 (21)
2	49/210 (23)	50/126 (40)	99/336 (29)
3	2/210 (1)	3/126 (2)	5/336 (1)
Whatever vaccinator recommends	46/210 (22)	52/126 (41)	98/336 (29)
Any no.	56/210 (27)	8/126 (6)	64/336 (19)
Reasons for allowing >2 injections^a^			
Limit no. of times away from work	2/104 (2)	0/63 (0)	2/167 (1)
It is better to receive all vaccines at once	14/104 (13)	2/63 (3)	16/167 (10)
Make sure child gets all vaccines	55/104 (53)	40/63 (63)	95/167 (57)
Doctor knows best	49/104 (47)	31/63 (49)	80/167 (48)
Reasons for allowing ≤2 injections^a^			
Avoid pain and discomfort	96/106 (91)	63/63 (100)	159/169 (94)
Too much for immune system	23/106 (22)	0/63 (0)	23/169 (14)
Preferences on how infants might receive 3 injections during immunization visits			
1 visit with 3 injections	81/210 (39)	34/126 (27)	115/336 (34)
2 visits with 1 or 2 injections during each visit	59/210 (28)	65/126 (52)	124/336 (37)
3 visits with 1 injection per visit	70/210 (33)	27/126 (21)	98/336 (29)

Abbreviation: EPI, Expanded Program on Immunization.

^a^ Multiple responses allowed.

When caregivers were asked about the maximum number of injections they were comfortable allowing their infant to receive during 1 immunization visit, 21% were comfortable with 1 injection; 29%, with 2 injections; 1%, with 3 injections; 29%, with whatever the vaccinator recommended; and 19%, with any number of injections (Table 6). When the question was rephrased so that caregivers were asked how they preferred to have 3 injectable vaccines administered to their infants, 34% preferred that all 3 injections be given during 1 immunization visit; 37% preferred that they be administered during 2 visits, with 2 injections at most in a single visit; and 29% preferred that they be administered during 3 visits, with 1 injection per visit. Among caregivers comfortable allowing >2 injections per visit, the reasons most commonly mentioned were to ensure that the child received all vaccines (57%) and that healthcare practitioners knew best (48%). Most caregivers (94%) who were not comfortable allowing their infant to receive >2 injections reported that they wanted to avoid pain for their child.

## DISCUSSION

This assessment after the introduction of IPV and PCV in the RI schedule in Bangladesh demonstrated that, overall, the introduction went smoothly, likely because of adequate planning and preparations by a robust immunization program. No negative effects on coverage were observed for pentavalent vaccine or OPV, based on analysis of administrative data and the community survey.

The major programmatic challenge identified was the presence of shortages and stock outs of IPV that began shortly after introduction and varied by district and CC. Records and interviews indicated that shortages were not caused by errors in vaccine management or storage in the district/CC offices or in health facilities but arose because dose requirements at the health facilities exceeded IPV availability nationwide. Consistent with concerns before IPV introduction, the use of 5-dose IPV vials at outreach vaccination sessions with small numbers of eligible children led to wastage above the expected 30% in most health facilities (80%). Immunization managers and vaccinators tried to cope with shortages by reducing the number of facilities administering IPV and the number of sessions during which IPV was administered, asking caregivers to wait or attend different sessions and often opening new vials only when >2 eligible children were present. These strategies may decrease wastage but may also increase the number of missed opportunities for IPV vaccination [12, 13]. The high proportion (32%) of caregivers who reported not knowing why their child did not receive IPV with the third dose of pentavalent vaccine, especially in areas with stock outs, suggests that some vaccinators might not have informed caregivers about IPV shortages.

Vaccinator and community surveys indicated that IPV was well accepted by health workers and caregivers. Although only 65% of infants received IPV as compared to 86% who received OPV3 and 87% who received Penta3, the major reported reasons for missing IPV were unavailability of vaccine and lack of awareness about IPV. None of the caregivers reported mistrust of IPV or concerns about receiving 2 polio vaccines (IPV and OPV3) at the same visit as being a reason for their children having missed IPV administration, which is supported by the high proportion of children who received OPV3 with or without IPV (86% in the community survey and 88% according to administrative data).

The Bangladesh EPI introduced a new immunization visit at 18 weeks of age to provide the third dose of PCV, out of concern for possible rejection by vaccinators or caregivers of the administration of 3 injectable vaccines in a single visit. Our assessment confirms the reluctance of vaccinators to administer 3 injections in 1 visit and their belief that most caregivers would prefer administration of only 1 or 2 injections per visit. However, similar to findings in other studies [14], vaccinators overestimated caregiver concerns because approximately 50% of caregivers said that they would be comfortable with 3 or any number of injections recommended by the vaccinator.

The high proportion of infants who received the first and second doses of PCV, as revealed in the community survey, suggests that the vaccine was also well accepted by caregivers; however, the low PCV3 coverage we observed is concerning. Because we did not ask about reasons for missing the third dose of PCV, we cannot confirm whether the observed dropout is related to caregivers’ unawareness of or challenges to attend the extra immunization visit or to delays in vaccination schedules. A WHO-led evaluation of PCV introduction, conducted in late November 2015, found that 30% of children who had received Penta3 did not receive PCV3, consistent with our results [15]. Further investigation is needed to confirm whether there is a persistent reduction in infants who receive PCV3 relative to OPV3 or Penta3, and its causes.

This assessment had several limitations. Because we used convenience sampling to identify districts/CCs and communities, the results cannot be generalized to all of Bangladesh. Our inability to interview caregivers who were not at home may also have introduced a bias in the community survey. Finally, issues that may take >3 months to manifest may not have been captured properly.

This assessment also has strengths. Inclusion of a community survey, which is usually not part of evaluations of vaccines after their introduction, provided a more comprehensive assessment of reasons for low coverage for IPV and allowed exploring discrepancies in perception and attitudes by health workers and caregivers. Collection of the same data from multiple sources also ensured the reliability of findings.

Despite the successful introduction of IPV in Bangladesh, insufficient vaccine supply is likely to limit its effect on population immunity against poliovirus. Following our assessment, Bangladesh received IPV vials suitable for use (ie, with vaccine vial monitors on the side), in accordance with the WHO's 28-day open-vial policy, which allows using a multidose vial for up to 28 days if the cold chain has been properly maintained [16]. Applying this policy would reduce significantly IPV vaccine wastage, but this alone will be insufficient to make up for further anticipated reductions in the supply of IPV during 2016–2017, because of a global shortage [17]. The limited IPV supply in Bangladesh could be further stretched by replacing the single 0.5-mL intramuscular dose given at 14 weeks with two 0.1-mL intradermal doses (fractional-dose IPV), as proposed by the WHO [18]. Two intradermal doses might be more immunogenic against type 2 poliovirus than a single intramuscular dose [18–20] and would allow vaccination of more than twice the number of infants with the same number of vials.
